# Factors associated with intention to implement SBI and SUD treatment: a survey of primary care clinicians in Texas enrolled in an online course

**DOI:** 10.1186/s12875-024-02427-z

**Published:** 2024-05-28

**Authors:** Alicia Kowalchuk, Tiffany G. Ostovar-Kermani, Kylie Schaper, Larissa Grigoryan, Jacqueline M. Hirth, Maria Carmenza Mejia, Kiara K. Spooner, Roger J. Zoorob

**Affiliations:** 1 Department of Family and Community Medicine, Baylor College of Medicine, 3701 Kirby Drive, suite 600, Houston, TX 77098 USA; 2 Department of Population Health and Social Medicine, Schmidt College of Medicine, Florida Atlantic University, 777 Glades Rd, Boca Raton, FL USA

**Keywords:** Substance use, Knowledge, Confidence, Intention, Primary care

## Abstract

**Background:**

Substance use disorder (SUD) presents a range of public health challenges and consequences. Despite the prevention potential of screening and brief intervention (SBI) in the primary care setting, implementation is low. The purpose of this study was to assess associations of primary care clinicians’ knowledge of SBI and SUD treatment, subjective norms, and perceived behavioral control with intention to incorporate SBI and SUD treatment into regular clinical practice.

**Methods:**

This online survey was administered to primary care clinicians who practice in Texas between March 1, 2021, and February 5, 2023. Survey questions were mapped to factors in the Theory of Planned Behavior and included measures of knowledge, subjective norms, and perceived behavioral controls related to SBI and SUD treatment. Intention to engage in SBI and SUD treatment was assessed as the outcome.

**Results:**

Of 645 participants included in this study, 59.5% were physicians. Knowledge was low, with less than half correctly reporting what was considered a standard drink (39.6%) and only 20% knew the correct number of alcoholic beverages considered risky drinking in 21-year-old non-pregnant women. Subjective norms, such as having colleagues within their practice support addressing SUDs, and perceived behavioral control such as having SUD screening routinized within clinic workflows, were positively associated with intention to implement SBI and SUD treatment in primary care settings.

**Conclusions:**

Modifying knowledge gaps, subjective norms, and perceived behavioral control requires a multipronged interventional approach that blends accessible clinician training with systemic workplace enhancements and a collective shift in professional norms.

**Supplementary Information:**

The online version contains supplementary material available at 10.1186/s12875-024-02427-z.

## Introduction

Risky substance use and substance use disorder (SUD) present a range of public health challenges and consequences at the individual, familial, community and societal levels. Adults with alcohol and other substance use disorders are disproportionately represented in U.S. primary care and emergency department patient populations [1, 2]. Thus, primary care settings provide an optimal platform for the prevention, screening and treatment of SUD over time, particularly as alcohol, tobacco, and other substances are associated with increased risks of preventable morbidity and mortality [3–5]. Screening and brief intervention (SBI) is one tool that can assess and address patients’ risky substance use in the primary care setting [6]. However, despite the US Preventive Services Task Force (USPSTF) recommending these practices combined with the benefits of SBI for substance use [6, 7], implementation in primary care settings is still infrequent [8–10].

To the knowledge of this study’s authors, there are a limited number of studies that provide a comprehensive assessment of primary care clinicians’ knowledge, attitudes, subjective norms, and intentions to use evidence-based SBI and SUD treatment in the primary care setting. Of the studies available, most are either centered on specific patient populations (such as adolescents and young adults) [11, 12], specific SUD types (i.e., opioid use disorder) [13], health professional students or trainees [14] or assess general perceptions, attitudes and self-efficacy related to screening practices or integration of AUD, nicotine use disorder, or opioid use disorder (OUD) in primary care [12]. Accordingly, more targeted investigations are needed to better understand clinician-level factors which may contribute to the implementation of evidence-based SUD prevention and treatment practices in primary care settings.

The purpose of this study was to assess the associations of knowledge, confidence, beliefs, and subjective norms with intention to implement SBI and SUD treatment among primary healthcare clinicians interested in enrolling in an online course on SBI and SUD treatment. This study used an in depth, theory driven^16^ exploration of factors associated with intent to implement screening and brief intervention (SBI) and SUD treatment services into practice.

## Methods

### Setting and respondents

This online survey was administered to health professionals recruited to participate in a free, interactive, asynchronous online course providing training on preventing and treating substance use disorders in primary care. The survey was administered at the time respondents registered for the course between March 1, 2021, and February 5, 2023. The targeted respondents were primary care clinicians in active clinical practice. Respondents were recruited through email invitations sent to professional networking and licensure organizations as well as academic institutions located in Texas. Organizations were identified by searching for local chapters of statewide organizations (supplemental Table 1). Advertisements were done in state-wide primary care conference programs, in-person promotion at medical clinics and offices, and in newsletters produced by statewide and local medical organizations that had a Texas audience. Potential respondents accessed the survey online and consented to participate. Those who agreed were gated to the survey questions. Respondents were not compensated for completing the survey. The study was approved by the Baylor College of Medicine Institutional Review Board (IRB).

For this study, all completed surveys from physicians, nurse practitioners, physician assistants, and medical fellows or residents were included. This evaluation excluded pharmacists, behavioral health clinicians, and students in medical and nursing programs because respondents in these categories would not be licensed to prescribe medications to treat SUDs. Respondents were also excluded if they were younger than 18 years old or practiced outside of Texas.

#### Demographic characteristics

Respondents were asked about their years in clinical practice, type of clinical practice, age group of practice patients, and whether any current practice patients have SUDs, along with respondents’ demographics including gender, race/ethnicity, and age. Years in practice were categorized as “<5 years,” “5–10 years,” and “11 + years.” Practice settings included “community health centers,” “private solo or group practices,” “hospital-based practices,” “academic practices,” or “other”. Specialties included “family medicine,” “internal medicine,” and “other”, which included addiction medicine, obstetrics/gynecology, pediatrics, psychiatry, preventive/occupational, and sports medicine. The patient age groups served were categorized as, “Pediatric patients only (< 18 years old),” “Adult patients only (18 + years old),” or “both adult and pediatric patients.”

#### Survey instrument

**Fig. 1:**
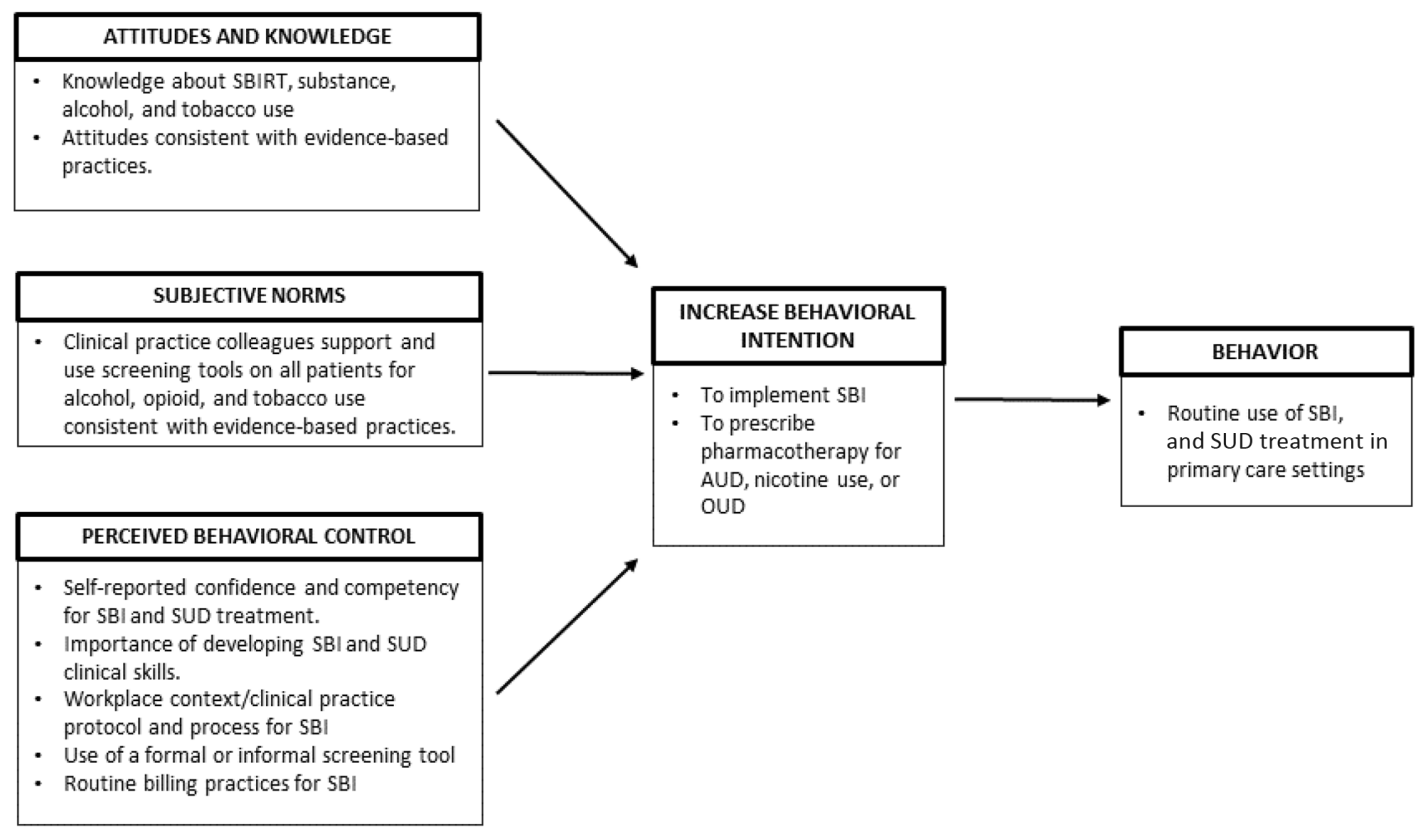
Theory of Planned Behavior model with elements integrated into survey for primary care clinicians

Survey questions were mapped to factors in the Theory of Planned Behavior (TPB), a well-validated conceptual model, to identify internal and external factors that influence intention to implement screening and brief intervention (SBI) and SUD treatment in clinical practice (Fig. 1) [15]. According to TPB, intention to perform the behavior of interest is determined by attitudes and beliefs about the behavior, the perceived subjective norms, and perceived behavioral control of an individual toward a particular behavior [16]. All survey questions are included in tables.

### Intention to implement SBI and SUD treatment

Intention to implement SBI and SUD treatment in clinical practice was assessed using 5-point Likert scale responses (ranging from ‘Extremely unlikely’ scored as 1 to ‘Extremely likely’ scored as 5) to 6 statements. The internal consistency of this measure was high (Cronbach’s alpha = 0.88) and scores were combined to create a continuous measure of intention. A binary variable using the 50th percentile for cutoff scoring was made. Thus, low intent was categorized for all respondents who scored 6–24 and high intent was categorized as all respondents who scored 25–30.

#### Knowledge/ attitudes

Knowledge components included 6 questions about alcohol, tobacco, and other SUD treatments. Responses were categorized as either “correct” or “incorrect.”

Respondent knowledge and attitudes were assessed using 6 questions from the 63 item Physician’s Competence in Substance Abuse Test (P-CSAT), a valid and reliable measure developed to assess clinical decision-making skills [17]. Six questions were used to assess agreement with statements about SBI and SUD treatment. Responses consisted of a 6-level scale, ranging from “strongly disagree” to “strongly agree” with no neutral options. Response categories were collapsed into 2 levels to reflect agreement or disagreement with each response.

#### Subjective norms

Subjective norms were measured using agreement with each of 3 questions: “In general, the other healthcare professionals I work with support screening all patients for alcohol use,” “In general, the other healthcare professionals I work with support screening all patients for opiate use,” and “In general, the other healthcare professionals I work with support screening all patients for tobacco use.” The responses consisted of a 6-point scale ranging from “strongly disagree” to “strongly agree” with no neutral responses. These questions were adapted from previously validated questions on identifying clinician barriers to evidence-based care [18]. Responses were scored as 1 for agreement, -1 for disagreement, and 0 for ‘not applicable’ or ‘don’t know’. Scores were summed to create a scale ranging between − 3 and 3. The internal consistency reliability of this scale was evaluated, with a Cronbach’s alpha of 0.82.

#### Perceived behavioral control

Perceived behavioral control was measured using a scale that included responses to 6 statements regarding the respondent’s confidence level in adopting SBI and SUD treatment practices, which were adapted from themes identified in a qualitative study assessing a program to implement SBI in an academic general medicine practice [19]. Responses consisted of a rating scale from 1 being the least confident to 10 being the most confident. Internal consistency for this scale was high (Cronbach’s alpha = 0.91), so item scores were combined and ranged from 6 to 60. Another question regarding the importance of developing clinical skills in SBI was analyzed separately.

### Statistical analyses

Bivariable analyses were conducted to examine associations between demographic information and other workplace context questions with the binary variable “intent to implement” SBI and SUD treatment in their practice using chi-square tests. The association between knowledge and intent as well as workplace factors and intent were assessed using binary logistic regression as these independent variables were unable to be constructed into a continuous scale variable.

To examine associations of subjective norms, confidence, and perceived behavioral control with intention to implement SBI and SUD treatment, linear regression models with intent as a continuous dependent variable were used. Linear regression models do not accurately reflect the impact of the categorical variables on the target variable, leading to potentially inaccurate predictions, thus 2 different types of models were used to evaluate associations.

All models controlled for demographic variables that were associated with the binary intention outcome in the bivariate analyses (alpha = 0.10). Final models were assessed for significant values using alpha = 0.05. Data were analyzed using SAS statistical software version 9.4 (Cary, NC).

## Results

During recruitment, 5,675 health care clinicians across Texas were contacted directly by email. Additionally, an undefined number of clinicians were contacted through advertisements and email sent by local or statewide medical profession networks and newsletters. The final survey included 1,583 individuals who viewed the survey after consenting. Of these, 645 met the inclusion criteria (40.7%).

### Respondent characteristics

Most respondents were family medicine clinicians (66.4%) and had MD/DO degrees (59.5%, Table 1). The sample included 36 residents/ fellows, of which 13 were in their first post graduate year, 10 in their second, and 11 in their third. No differences in binary outcome for intent were found for demographic variables except for specialty and gender. Intention to implement SBI and SUD treatment was associated with specialty with 36% of Family Medicine clinicians having high intent to implement compared to internal medicine (28.7%) and other primary care (24.6%) practitioners (*p* < 0.05).

**Table 1:** Association between healthcare professional characteristics and intention to implement SBI and SUD treatment among primary care clinicians in Texas (*N* = 645)

	Total *n* = 645 *n* (%)	Low intent (< 25) *n* = 434 *n* (%)^a^	High intent (≥ 25) *n* = 211 *n* (%)^a^	*P*-value^b^
**Race/ ethnicity**				
Hispanic	94 (14.6%)	62 (66.0%)	32 (34.0%)	0.90
Non-Hispanic Asian	143 (22.2%)	98 (68.5%)	45 (31.5%)	
Non-Hispanic Black/African American	52 (8.1%)	37 (71.2%)	15 (28.9%)	
Non-Hispanic White	252 (39.1%)	165 (65.5%)	87 (34.5%)	
Non-Hispanic Other^c^	104 (16.1%)	72 (69.2%)	32 (30.8%)	
**How would you describe your gender?**				
Male	209 (32.4%)	152 (72.7%)	57 (27.3%)	**0.07**
Female	387 (60.0%)	247 (63.8%)	140 (36.2%)	
Prefer not to answer	49 (7.6%)	35 (71.4%)	14 (28.6%)	
**Clinician type**				
MD/DO	384 (59.5%)	251 (65.4%)	133 (34.6%)	0.21
NP/PA	225 (34.9%)	161 (71.6%)	64 (28.4%)	
Resident/Fellow	36 (5.6%)	22 (61.1%)	14 (38.9%)	
**Years practicing medicine or health specialty**				
< 5 years	191 (29.6%)	127 (66.5%)	64 (33.5%)	0.15
5–10 years	154 (23.9%)	95 (61.7%)	59 (38.3%)	
11 + years	300 (46.5%)	212 (70.7%)	88 (29.3%)	
**Which type of organization or practice best describes your current workplace?**				
Community Health Center	146 (22.6%)	98 (67.1%)	48 (32.9%)	0.64
Private Practice Group/Solo	208 (32.3%)	133 (63.9%)	75 (36.1%)	
Hospital-based Practice	119 (18.5%)	86 (72.3%)	33 (27.7%)	
Academic Practice	152 (23.6%)	104 (68.4%)	48 (31.6%)	
Other^d^	20 (3.1%)	13 (65.0%)	7 (35.0%)	
**Specialty**				
Family Medicine	428 (66.4%)	274 (64.0%)	154 (36.0%)	**0.04**
Internal Medicine	87 (13.5%)	62 (71.3%)	25 (28.7%)	
Other^e^	130 (20.2%)	98 (75.4%)	32 (24.6%)	
**Age Groups of Patients Treated**				
Pediatric Only	12 (1.9%)	11 (91.7%)	1 (8.3%)	0.15
Adult Only	260 (40.3%)	178 (68.5%)	82 (31.5%)	
Pediatric & Adult	373 (57.8%)	245 (65.7%)	128 (34.3%)	

^a^ Scale representing intent consisted of responses to 6 questions with 5 point-Likert scale responses. Possible scores ranged from 6–30. Univariate analyses revealed that the 50% cutoff point was a score of 24 which was used to create a binary outcome, including Low intent (score of 6–24) and high intent (score of 25–30)

^b^ The p-value was calculated using chi-square tests to examine associations between clinician characteristics and level of intent to implement SBI. Significance was calculated using alpha = 0.10

^c^ Respondents who selected the other category consisted of non-Hispanic: American-Indian or Alaskan native, Native Hawaiian or Pacific Islander, multi-racial, prefer not to answer

^d^ Other workplace consists of those who are in school, corrections, occupational health, or do not have a current regular practice they work at, but still treat patients (e.g., volunteer, clinical work, locums)

^e^ Other specialty consists of those who specialize in: Addiction medicine, Obstetrics/Gynecology, Pediatrics, Psychiatry, Preventive/ Occupational or Sports Medicine

#### Knowledge/ attitudes

Responding correctly to knowledge questions about the safe level of alcohol use during pregnancy (adjusted odds ratio (aOR): 1.08, 95% CI: 0.72–1.62) and what is considered a standard drink were not associated with intent (aOR: 1.20, 95% CI: 0.86–1.68; Table 2). Less than half of respondents knew what was considered a standard drink (39.6%) and only 20% knew the correct number of alcoholic beverages considered risky drinking in 21-year-old non-pregnant women. Similarly, only 23% knew of an evidence-based screening tool for adolescent substance use. Knowledge of the number of drinks considered risky drinking was associated with 1.5 times the odds (95% CI: 1.05–2.28) of having high intent. Knowledge about the proper evidence-based screening tools to screen for SUD in primary care was associated with 2 times the odds (95% CI: 1.38–2.91) of having high intent. However, knowing the first line medication for tobacco cessation and which screening tools were validated to screen for adolescent substance use were not significantly associated with intent.

**Table 2:** Binary logistic regression assessing the association between knowledge/ attitude and level of intention to Implement SBI and SUD treatment in primary care setting among practicing primary care clinicians in Texas (high vs. low intent, *N* = 645)

Knowledge Scores (% correct)^a^	Total *n* (%)	Low intent (< 25) *n* = 434 *n* (%) ^b^	High intent (≥ 25) *n* = 211 *n* (%) ^b^	aOR (95% CI)^c, d^
Advice you would give to pregnant patient about how much alcohol is safe during pregnancy ^a^	502 (77.8%)	336 (77.4%)	166 (78.7%)	1.08 (0.72–1.62)
Which is considered a standard drink ^a^	264 (40.9%)	172 (39.6%)	92 (43.6%)	1.20 (0.86–1.68)
Risky drinking in 21-yr old non-pregnant women (per week) ^a^	147 (22.8%)	88 (20.3%)	59 (28.0%)	**1.55 (1.05–2.28)**
Evidence-based screening tool(s) used for SUD in primary care ^a^	418 (64.8%)	259 (59.7%)	159 (75.4%)	**2.01 (1.38–2.91)**
First-line medication to treat tobacco cessation ^a^	357 (55.3%)	230 (53%)	127 (60.2%)	1.29 (0.92–1.82)
Validated evidence-based screen for adolescent substance use ^a^	145 (22.5%)	99 (22.8%)	46 (21.8%)	0.96 (0.64–1.43)
**There is good evidence that primary care physicians can use brief interventions to decrease alcohol use in patients who drink at excessive levels.**				
Disagree (referent)	48 (7.44%)	37 (8.5%)	11 (5.2%)	1.62 (0.80–3.26)
Agree	597 (92.56%)	397 (91.5%)	200 (94.8%)	
**If a patient fails to respond to acamprosate to achieve abstinence from alcohol, naltrexone will show no benefit and should not be used.**				
Disagree	434 (67.29%)	279 (64.3%)	155 (73.5%)	**1.52 (1.05–2.20)**
Agree (referent)	211 (32.71%)	155 (35.7%)	56 (26.5%)	
**Opioid cravings can be treated in an office setting with buprenorphine.**				
Disagree (referent)	171 (26.51%)	123 (28.3%)	48 (22.8%)	1.34 (0.91–1.98)
Agree	474 (73.49%)	311 (71.7%)	163 (77.2%)	
**A local pharmacist contacts you because one of your patients has also been receiving prescriptions for hydrocodone from two other doctors. The most appropriate management is to tell the pharmacist to cancel the prescription and to discharge the patient from your practice.**				
Disagree	410 (63.57%)	263 (60.6%)	147 (69.7%)	**1.52 (1.06–2.16)**
Agree (referent)	235 (36.43%)	171 (39.4%)	64 (30.3%)	
**A 34-year-old patient is not ready to stop drinking. The most appropriate next step, using motivational interviewing, is to forcefully confront him with the likely health consequences of continued alcohol use.**				
Disagree	475 (73.64%)	307 (70.7%)	168 (79.6%)	**1.50 (1.01–2.24)**
Agree (referent)	170 (26.36%)	127 (29.3%)	43 (20.4%)	
**A person is more likely to be successful if they focus on quitting both alcohol and nicotine at once rather than one at a time.**				
Disagree	427 (66.2%)	284 (65.4%)	143 (67.8%)	0.95 (0.67–1.36)
Agree (referent)	218 (33.8%)	150 (34.6%)	68 (32.2%)	

^a^ The percents shown are for the percent who responded correctly to the knowledge questions

^b^ Scale representing intent consisted of responses to 6 questions with 5 point-Likert scale responses. Possible scores ranged from 6–30. Univariate analyses revealed that the 50% cutoff point was a score of 24 which was used to create a binary outcome, including Low intent (score of 6–24) and high intent (score of 25–30). Column percents shown

^c^aOR = Odds ratio adjusted for gender and clinician specialty. 95% CI = 95% confidence interval

^d^aORs compared correct responses to incorrect responses or attitudes consistent with evidence-based recommendations to those not consistent with evidence-based recommendations

Questions that assessed attitudes about utilization of different tools to intervene on patients at risk of SUDs in the office setting were also used to determine their association with intention (Table 2). Overall, attitudes were consistent with evidence-based practice. For example, most respondents agreed that there is good evidence that primary care physicians can use brief interventions to decrease alcohol use (91.5%). More than half disagreed that patients failing to respond to acamprosate will show no benefit from naltrexone (64.3%).

Intent to implement SBI and SUD treatment in multivariable binary logistic regression models was positively associated with guideline-consistent attitudes regarding patients who fail to respond to acamprosate to achieve abstinence from alcohol (aOR: 1.52, 95% CI: 1.05–2.20; Table 2). Disagreeing with telling a pharmacist to cancel a prescription and discharging a patient from a practice in response to patient receiving multiple prescriptions for hydrocodone had 1.5 times the odds (95% CI: 1.06–2.16) of having high intent. Disagreement with the practice of forcefully confronting a patient with likely health consequences if they are not ready to stop drinking was associated with 1.5 times the odds (95% CI: 1.01–2.24) of having high intent. Other questions on attitudes were not associated with intent after controlling for gender and clinician specialty. Family medicine clinicians had higher odds of responding correctly to knowledge and having attitudes more consistent with evidence-based practices compared to “other” primary care clinicians (supplemental Table 2). For example, family medicine was more likely to know what constitutes risky drinking in a 21-year-old non-pregnant woman (aOR: 1.79; 95% CI: 1.14–2.81) and to have evidence-based recommendation consistent attitudes regarding treating opioid cravings in an office setting with buprenorphine compared to “other” types of clinicians (aOR: 1.72, 95% CI: 1.10–2.70).

#### Subjective norms

Subjective norms were positively associated with intention to implement SBI and SUD treatment in primary care settings (Table 3). Intention was high among respondents with scores indicating other healthcare clinicians supported screening all patients for alcohol use (*p* < 0.001), nicotine use (*p* < 0.001), and opioid use (*p* < 0.05).

**Table 3:** Association of subjective norms with intention to implement SBI and SUD treatment among primary care clinicians in Texas (*N* = 645)

	β* (95% CI) ^a^	*p*-value
Subjective Norms/Intervention: Agree that other healthcare professionals I work with support screening all patients for *alcohol* use	1.07 (0.52–1.62)	**< 0.001**
Subjective Norms/Intervention: Agree that other healthcare professionals I work with support screening all patients for *tobacco* use	0.90 (0.39–1.40)	**< 0.001**
Subjective Norms/Intervention: Agree that other healthcare professionals I work with support screening all patients for *opioid* use	0.73 (0.08–1.39)	**0.03**

^a^Linear regression models controlled for respondent gender and clinician specialty. Subjective norms were continuous and ranged from − 1 to 1

#### Perceived behavioral control

Higher confidence scores in clinical skills was positively associated with intention to implement SBI and SUD treatment (*p* < 0.001; Table 4). Respondents who were more confident about the importance of developing their clinical skills for SBI had higher intent to implement SBI and SUD treatment (*p* < 0.001). Workplace practices were also associated with intent to implement SBI and SUD treatment. There was an increase of almost 6 times the odds among those who reported their practice screens annually (95% CI: 1.39–25.77) or at each patient visit (95% CI: 1.124–26.06) with high intent to implement SBI and SUD treatment compared with those who never screen. A positive association between clinicians who reported that their practice has a protocol and process to screen patients for nicotine, alcohol, or opioid use and high intent to implement was found (aOR: 1.64, 95% CI: 1.17–2.31). A positive association between screening followed by an intervention and having high intent to implement was also found (aOR: 1.97, 95% CI: 1.29–3.01).

**Table 4:** Association of perceived behavioral control (confidence and workplace practices) with intention to implement SBI and SUD treatment (*N* = 645)

	Mean (stdev)	Low intent (< 25) *n* = 434 mean (stdev)	High intent (≥ 25) *n* = 211 mean (stdev)	β (95% CI)
**Confidence score** (range 6–60) ^a^	32.2 (10.7)	30.10 (9.6)	36.65 (11.5)	**0.183 (0.153–0.213)**
**How important is it for you to develop your clinical skills regarding screening, brief intervention, and referral to treatment (SBIRT) for substance use disorders (including prescription drug misuse)?** (range 1–10)	8.2 (1.9)	7.7 (1.9)	9.1 (1.4)	**1.09 (0.919–1.263)**
**Workplace context**	**N (%)**	**Low intent (< 25)** *n* = 434 **n (%)** ^b^	**High intent (≥ 25)** *n* = 211 **n (%)** ^b^	**aOR (95% CI)** ^c, d^
**When do you (or someone in your practice) ask your patients or their parents/caregivers about their alcohol or opiate use?**				
Annually, At Each Visit	536 (83.1%)	352 (81.1%)	184 (87.2%)	**5.98 (1.39–25.77)**
Other, When I think it is Indicated, First Visit	82 (12.7%)	57 (13.1%)	25 (11.8%)	**5.68 (1.24–26.06)**
Never	27 (4.19%)	25 (5.8%)	2 (0.9%)	Referent
**My practice has a protocol and consistent process to screen or obtain information from patients about their nicotine, alcohol, or opioid use**:				
Yes	333 (51.6%)	205 (47.2%)	128 (61.7%)	**1.64 (1.17–2.31)**
None Currently/Don’t Know/Not applicable	312 (48.4%)	229 (52.8%)	83 (39.3%)	Referent
**What does initial patient screening for nicotine, alcohol and/or opiate use consist of in your practice setting?**				
Informal questions and/or a formal screening tool/validated instrument	317 (95.2%)	196 (45.2%)	121 (57.3%)	**1.56 (1.11–2.18)**
No Screening/ I don’t know	328 (50.9%0	238 (54.8%)	90 (42.7%)	Referent
**Is screening followed by some type of intervention in your practice setting? (n = 645)** ^**b**^				
All patients are given educational materials/information on substance use and health	85 (13.2%)	46 (10.6%)	39 (18.5%)	**1.83 (1.15–2.92)**
No educational materials given	560 (86.82%)	388 (89.4%)	172 (81.5%)	Referent
Patients who screen positive for substance use disorder are asked follow-up questions and provided with brief counseling	158 (24.5%)	90 (20.7%)	68 (32.2%)	**1.75 (1.20–2.55)**
Patients not asked follow-up questions or provided with brief counseling	487 (75.5%)	344 (79.3%)	143 (67.8%)	Referent
Patients who screen positive for substance use disorder are asked follow-up questions and provided with additional resources (e.g., a list of treatment and/or counseling services in the community)	109 (16.9%)	58 (13.4%)	51 (24.2%)	**1.97 (1.29–3.01)**
Patients not asked follow-up questions or provided with additional resources	536 (83.1%)	376 (86.6%)	160 (75.8%)	Referent
**Does your practice bill for screening and brief intervention services?**				
Yes	121 (18.8%)	71 (16.4%)	50 (23.7%)	1.51 (1.00–2.28)
No/Not Sure	524 (81.2%)	363 (83.6%)	161 (76.3%)	Referent

^a^Linear regression models controlled for respondent gender and clinician specialty. Confidence included the sum of responses to 6 total questions with sliding scales ranging from 1 to 10 for a total possible range of 6–60

^b^ Column percents shown

^c^ Scale representing intent consisted of responses to 6 questions with 5 point-Likert scale responses. Possible scores ranged from 6–30. Univariate analyses revealed that the 50% cutoff point was a score of 24 which was used to create a binary outcome, including Low intent (score of 6–24) and high intent (score of 25–30). Binary logistic regression models controlled for respondent gender and clinician specialty. The referent level for the dependent variable was low intent

^d^ Bolded values indicate significance at *p* < 0.05

## Discussion

Using the TPB model, multiple predictors of intention to implement SBI and SUD treatment into primary care practice were found among clinicians interested in enrolling in an online course on the topics. Specifically, knowledge about risky drinking limits, attitudes consistent with evidence-based practices, subjective norms such as having colleagues in their practice supportive of addressing substance use and perceived behavioral control such as having substance use screening routinized within clinic workflows were associated with higher intention. Additionally, the family medicine specialty was associated with greater knowledge of, higher intention for, and favorable attitudes toward evidence-based practices.

The USPSTF aims to foster a more proactive and effective approach to addressing substance use by shifting the focus from preventive counseling to improving diagnosis and treatment of SUD. This recommendation encourages healthcare clinicians to play a vital role in identifying and supporting individuals with substance use disorders, ultimately leading to better health outcomes and a more comprehensive approach to healthcare delivery. However, this survey of more than 600 primary care professionals from various settings found that knowledge about evidence-based SBI and treatment of SUD was poor. Particularly concerning is the finding that less than half of the respondents knew what constituted a standard alcoholic drink, and only one-fifth could correctly identify the risky drinking limits for young adult women. This lack of foundational knowledge could impede effective screening and intervention for patients at risk of AUDs. Interestingly, while general knowledge didn’t strongly correlate with implementation intention, specific knowledge areas did, indicating that a certain knowledge threshold enhances implementation intent. In this study, clinicians with better knowledge of evidence-based screening tools for SUD in primary care were more likely to have a high intent to implement these tools. Primary care clinicians ascribe their infrequent implementation of SBI and SUD treatment to knowledge and skill related factors, such as limited familiarity with current recommendations, along with having an insufficient skillset confidence to implement [19, 20]. This suggests that improving knowledge might enhance the uptake of these practices. This study indicates that increased knowledge is associated with increased intention to implement SBI and SUD treatment among primary care clinicians who did not necessarily have specialized training. In the context of the Mainstreaming Addiction Treatment Act [21], which allows the prescription of evidence-based treatment for OUD with standard DEA registration, this study illuminated the significance of empowering primary care clinicians without specialized training.

Respondents’ attitudes generally aligned with evidence-based recommendations. The majority recognized the efficacy of brief interventions in reducing alcohol consumption and of medications to treat SUDs and opposed practices like forceful confrontation of patients or discontinuation of care for those with multiple prescriptions. However, those with attitudes inconsistent with evidence-based recommendations had lower intent, and represented up to a third of respondents, suggesting interventions in this domain may still be impactful. Findings also highlighted knowledge and attitude disparities across specialties and gender, suggesting the potential for targeted intervention strategies.

The role of subjective norms, or the perception of what is deemed acceptable or standard practice by peers, cannot be understated. This study demonstrated a strong association between the perception of peer endorsement for screening and a clinician’s intent to implement such practices. This association underlines the importance of fostering a collective professional culture that prioritizes SBI and SUD treatment, suggesting that change may not only be driven from individual knowledge and beliefs but also through shared norms and practices.

Perceived behavioral control emerged as an influential factor; clinicians with a higher confidence in their abilities and who perceived supportive workplace environments exhibited stronger intent for implementation. Key workplace factors—such as the frequency of patient screenings for alcohol and opiate use, the existence of protocols for SUD screening, the availability of screening tools, and the implementation of follow-up interventions—were positively associated with high intent to implement SBI and SUD treatment. These findings align with existing research emphasizing the importance of organizational support and the integration of practical, evidence-based protocols into daily practices for the successful adoption and sustainability of health interventions [22, 23].

Addressing structural barriers, including time constraints for provision of preventive care [24, 25], lack of resources, access to specialized counseling staff and practice-wide adoption of integrated mental health models, insurance restrictions, need for training opportunities, and workflow and practice management demands, remains critical [26–28]. Training clinicians to bill for SBI services and making training accessible and adaptable to time challenges could address some of the barriers to implementation. Many programs target improving knowledge, but workplace factors (such as how often patients are asked about alcohol or opiate use, whether their practice has a protocol to screen patients for SUDs, if a screening tool is available in the practice, and whether there is an intervention followed in the clinic) had strong associations with intention to implement. Future interventions may need to ensure that there is sufficient support in the workplace to integrate SBI and SUD treatment into primary care settings.

Programmatic offerings that increase specialized knowledge, enhance clinician confidence, align subjective norms, and guide practice and systems level changes needed to support SBI and SUD treatment workflows may normalize adoption. For example, in promoting SBI in primary care, it was found that 2 out of 4 facilitators, including having clinic champions and effecting systemic change, improved SBI implementation during the initial rollout of the program in the US [29]. Best practices such as ongoing SBI training, having a practice champion to encourage buy-in and engagement, aligning SBI with practice workflow and utilizing an interprofessional team, can help improve the implementation and sustainability of SBI in primary care practices, all of which may work through increasing perceived behavioral control [30].

A study of an organization readiness implementation intervention utilized methods aimed to increase the competency and confidence of providing SUD treatment among primary care clinicians. In addition, the program implemented processes and procedures to improve use of SUD treatment among patients [31]. Interventions included selecting and training champions, pre-testing and adapting protocols, providing training to clinicians and staff, and provide technical assistance. Although these interventions were well-received, they were associated with small, but not significant, increases in intention to treat patients after they were implemented, which may have been partially due to low power to detect differences (*N* = 69) [31]. These findings suggested that comprehensive, theoretically grounded organizational strategies might not fully enhance integration of medication treatment of SUDs in primary care. This emphasizes the need for further endeavors to equip primary care clinicians, especially regarding medications for treating OUD. Another small study of an educational intervention to increase uptake of Naltrexone among patients with AUD in a residential SUD treatment facility found that a brief online educational intervention increased use of the drug after the intervention, although the study lacked power to determine significance of the finding [32]. However, evidence showing the effect of education on this topic is sparse. These studies suggest the potential utility of TPB principles in increasing uptake of SBI and SUD treatment.

In this study, family medicine practitioners had higher odds of responding correctly to knowledge questions and positive attitudes toward implementation of SBI and SUD treatment compared to Other types of physicians and internists. This suggests targeting family medicine practices for implementation efforts or further studying factors associated with specialty differences may be worthwhile. Although many clinicians may report screening their patients for SUDs, offering brief intervention and treatment options are not as common, even among patients who need quick access to treatment [33]. Clinicians acknowledge that there are barriers to offering these services, such as competing priorities and better leadership in organizing to promote efforts to effectively intervene and provide treatment to their patients [28]. The data from this study demonstrated that there is interest in implementing these services in primary care. Therefore, providing an educational intervention may only be one step toward improving provision of these services routinely into primary care settings. For example, offering behavioral or psychological services through a licensed clinician within the same clinic has been offered as one possible method of increasing intervention and treatment services [28].

Respondents consisted of clinicians across the State of Texas who enrolled in an online SBI and SUD course. While this does limit this study’s generalizability to clinicians across the US, this sample consisted of a diverse set of clinicians practicing in both urban and rural regions and in a variety of practice settings. Further, although recruitment was aimed broadly at all clinicians across Texas, there was a possibility of selection bias in the types of clinicians (profession and medical specialty) who had the opportunity to view ads for this program or special interest on the topic. Another limitation is that respondents were recruited through an invitation to participate in an online course that addressed substance use prevention and treatment in the primary care setting. We used online recruitment due to limitations posed by the COVID-19 pandemic which limited in-person meetings. Since we could not assess the number of people who read emails or who viewed advertisements for this program, we could not calculate the overall response rate for this study. Although respondents took the survey before accessing course content, there may have been response bias with clinicians who were interested in implementation or who had more knowledge about SUDs included in the sample. However, even with this bias, it was demonstrated that there is still a lack of knowledge and use of SBI and SUD treatment interventions. Thus, the general knowledge and intention is likely to be lower among those who did not participate after invitation if they did not have previous training. Finally, this study was cross-sectional in design and thus, causation could not be inferred by the results.

## Conclusion: implications for behavioral health

While there is greater awareness and acceptance of the importance of SBI and SUD treatment in primary care, their inconsistent application arises from a myriad of factors, ranging from knowledge gaps to practice constraints. Employing best practices could substantially enhance the incorporation and longevity of SUD interventions in primary care settings, improving care for a wider patient population. One of the strongest factors associated with intention to implement was workplace context, including having established protocols for screening and having some type of intervention plan in place, indicating need for additional components to improve implementation of SBI and SUD treatment. Addressing these challenges requires a multipronged approach, blending individual clinician training with systemic workplace enhancements and a collective shift in professional norms. As the healthcare landscape continues to evolve, the responsibility is on all stakeholders to ensure that the primary care sector is equipped to positively impact substance use in their patients.

### Electronic supplementary material

Below is the link to the electronic supplementary material.

Supplementary Material 1

Supplementary Material 2

## Data Availability

No datasets were generated or analysed during the current study.
