# Supplementary material for: Factors associated with intention to implement SBI and SUD treatment: a survey of primary care clinicians in Texas enrolled in an online course
Source: BMC Prim Care. 2024 May 28;25:192. doi: 10.1186/s12875-024-02427-z (PMC11134618; doi:10.1186/s12875-024-02427-z)
Supplement: Supplementary file 1 — Supplementary Material 1 [file 12875_2024_2427_MOESM1_ESM.docx]

Supplemental Table 1. Organization contacts and methods of advertising to Texas healthcare clinicians

| **Organization** | **Method of Contact with organization** | **Method of contact from organization to its members** | **Type of contact** |
| --- | --- | --- | --- |
| Alamo Chapter of TAFP | Email |  | Organization Contact |
| Alumni Email for BCM Physician Assistants | Email |  | School Contact |
| Alumni for University of North Texas Health Science Center at Fort Worth | Email |  | School Contact |
| Alumni of UTMB PA, FNP, and MD | Email |  | School Contact |
| Amarillo Area PA Society | Email |  | Organization Contact |
| Amarillo Chapter TAAP | Email |  | Organization Contact |
| American College of Physicians - Texas Chapter | Email |  | Organization Contact |
| Austin Area PA Society | Email |  | Organization Contact |
| Austin Branch of Texas Society of Psychiatric Physicians | Email |  | Organization Contact |
| Austin Chapter TAAP | Email |  | Organization Contact |
| Austin Psychoanalytic Association | Email |  | Organization Contact |
| Behavioral Health Advocates of Texas | Email |  | Organization Contact |
| Bexar County Branch of Texas Society of Psychiatric Physicians | Email |  | Organization Contact |
| Bexar County Medical Society Alliance | Email |  | Organization Contact |
| Big Country County Medical Society Alliance | Email |  | Organization Contact |
| Brazos Valley Chapter TAAP | Email |  | Organization Contact |
| Brazos Valley Nurse Practitioner Association | Form on Website |  | Organization Contact |
| Brazos-Robertson County Medical Alliance | Form on Website | Email to residents | Organization Contact |
| Cameron Willacy County Medical Society | Email | email to members | Organization Contact |
| Carl R. Darnall AMC (Fort Hood) | Email |  | School Contact |
| Carl R. Darnall Army Medical Center | Email |  | School Contact |
| Central Texas Association of Black Social Workers | Email |  | Organization Contact |
| Christus Santa Rosa Health Care | Email |  | School Contact |
| Commerce Chapter TAAP | Email |  | Organization Contact |
| Conroe Education Foundation Fam Med Res Program | Email |  | School Contact |
| Dallas Chapter of TAAP | Email |  | Organization Contact |
| Dallas Chapter of TAFP | Email |  | Organization Contact |
| Dallas County Medical Society Alliance Foundation | Email |  | Organization Contact |
| Dallas/Fort Worth Area PA Society | Email |  | Organization Contact |
| District 7 TNA | Email |  | Organization Contact |
| East Texas Nurse Practitioners. | Form on Website | Promote on website & at monthly meetings | Organization Contact |
| El Paso Chapter TAAP | Email |  | Organization Contact |
| Fam Med Christus Spohn Memorial Hospital Fam Res Program | Email |  | School Contact |
| Fam Med es Program, HCA Gulf Coast Houston | Email |  | School Contact |
| Fam Med Res Program Baylor Med Ctr at Garland | Email |  | School Contact |
| Fam Med Res Program, Plaza Med Ctr, Ft Worth | Email |  | School Contact |
| Fam Med Res Program, Texas A&M, Temple BSW Hospital | Email |  | School Contact |
| Fam Med Res Program, Tx A&M, BSW Med Ctr, Round Rock | Email |  | School Contact |
| Fam Res Med, Valley Baptist Med Ctr (RGV) | Email |  | School Contact |
| Fort Worth Chapter of TAAP | Email |  | Organization Contact |
| Fort Worth Region Nurse Practitioners | Form on Website |  | Organization Contact |
| Galveston Chapter of TAFP | Email |  | Organization Contact |
| Galveston-Brazoria Branch of Texas Society of Psychiatric Physicians | Email |  | Organization Contact |
| GME Residency Program Coordinator - | Email |  | School Contact |
| Hardin-Simmons University PA Program | Email |  | Organization Contact |
| Harris County Chapter of TAFP | Email | Post on website; email to members | Organization Contact |
| Heart of Texas Chapter TAFP | Email |  | Organization Contact |
| Hidalgo-Star County Medical Society | Email |  | Organization Contact |
| Houston ABSW | Email |  | Organization Contact |
| Houston Area Nurse Practitioners | Email |  | Organization Contact |
| Houston Area PA Society | Email |  | Organization Contact |
| Houston Chapter TAAP | Email |  | Organization Contact |
| Houston of Texas Society of Psychiatric Physicians | Email |  | Organization Contact |
| Jefferson CMS | Email |  | Organization Contact |
| John Peter Smith Hosp, Ft Worth | Email |  | School Contact |
| Laredo Medical Center | Email |  | School Contact |
| Lubbock County Medical Society Alliance | Email |  | Organization Contact |
| Medical City Arlington | Email |  | School Contact |
| Medical City Fort Worth | Email |  | School Contact |
| Medical City Fort Worth (Univ of N Texas) | Email |  | School Contact |
| Memorial Family Medicine Residency Program, Houston | Email |  | School Contact |
| Mental Health America of Texas | Email |  | Organization Contact |
| Methodist Health System Dallas | Email |  | School Contact |
| Metro West North Texas Nurse Practitioners | Email |  | Organization Contact |
| Midland County Medical Society | Email |  | Organization Contact |
| North Harris Montgomery Advanced Practice Nurse Society | Email |  | Organization Contact |
| North Texas ABSW | Email |  | Organization Contact |
| Nueces County Medical Society | Email |  | Organization Contact |
| Panhandle Branch of Texas Society of Psychiatric Physicians | Email |  | Organization Contact |
| Potter-Randall County Medical Society Alliance | Email |  | Organization Contact |
| President of Permian Basin Area PA Society | Email |  | Organization Contact |
| President of Texas Gulf Coast PA Society | Email |  | Organization Contact |
| Rio Grande Valley Area PA Society | Email | In presentation to members | Organization Contact |
| Rio Grande Valley TAAP | Email |  | Organization Contact |
| Sam Houston State University College of Osteopathic Medicine/Baptist Hospitals of Southeast Texas | Email |  | School Contact |
| San Antonio Area PA Society | Email |  | Organization Contact |
| San Antonio Chapter of TAAP | Email |  | Organization Contact |
| San Antonio Metro ABSW | Email |  | Organization Contact |
| Smith County Medical Society | Form on Website |  | Organization Contact |
| South Plains Chapter TAFP | Email |  | Organization Contact |
| South Plains Nurse Practitioners Association (SPNPA) | Form on Website |  | Organization Contact |
| Southeast Texas Nurse Practitioners | Form on Website |  | Organization Contact |
| Tarrant Branch of Texas Society of Psychiatric Physicians | Email |  | Organization Contact |
| Tarrant County Chapter TAFP | Email | Email to members | Organization Contact |
| Tarrant County Medical Society Alliance | Email |  | Organization Contact |
| Texas A&M College Station Dept of Fam Residency Program | Email |  | School Contact |
| Texas A&M Family Medicine Clerkship - College Station | Email |  | School Contact |
| Texas A&M Family Medicine Clerkship - Dallas | Email |  | School Contact |
| Texas A&M Family Medicine Clerkship - Temple | Email |  | School Contact |
| Texas A&M Internal Medicine Clerkship - College Station | Email |  | School Contact |
| Texas A&M Internal Medicine Clerkship - Dallas | Email |  | School Contact |
| Texas A&M Internal Medicine Clerkship - Temple | Email | Email to members | School Contact |
| Texas A&M Medicine Program Manager | Email |  | Organization Contact |
| Texas A&M Psychiatry Clerkship - College Station | Email |  | School Contact |
| Texas A&M Psychiatry Clerkship - Dallas | Email |  | School Contact |
| Texas A&M Psychiatry Clerkship - Temple | Email |  | School Contact |
| Texas A&M Rural Health Institute | Email |  | School Contact |
| Texas Academy of Family Physicians | Email | Listed on "Other CME" page; Sent in News Now blast | Organization Contact |
| Texas Association of Community Health Centers | Email | Email to members | Organization Contact |
| Texas Association of Rural Health Clinics | Email |  | Organization Contact |
| Texas Association of Behavior Specialists | Email |  | Organization Contact |
| Texas Association of Rural Health Clinics | Email |  | Organization Contact |
| Texas Gulf Coast Physician Assistant Association | Email | Email to Members | Organization Contact |
| Texas Health Institute | Email |  | Organization Contact |
| Texas Health Physicians Group | Form on Website |  | Organization Contact |
| Texas Nurse Practitioners | Form on Website | Advertisement in TNP Pulse (x2) | Organization Contact |
| Texas Osteopathic Medical Association | Email |  | Organization Contact |
| Texas Primary Care Consortium | Email | Include in Newsletter to members | Organization Contact |
| Texas Primary Care Office (TPCO) | Email |  | Organization Contact |
| Texas Society of Addiction Medicine | Form on Website |  | Organization Contact |
| Texas Society of Psychiatric Physicians | Email |  | Organization Contact |
| Texas Southern University ABSW | Email |  | Organization Contact |
| Texas Tech El Paso Core Residency | Email |  | School Contact |
| Texas Tech Lubbock | Email |  | School Contact |
| Texas Tech Physicians of El Paso | Email |  | School Contact |
| Texas Tech, Permian Basin | Email |  | School Contact |
| The Center for Healthcare Services | Form on Website |  | Organization Contact |
| The Hill Country Nurse Practitioners Association | Form on Website |  | Organization Contact |
| The Panhandle Nurse Practitioners Association | Form on Website |  | Organization Contact |
| The Victoria Area Nurse Practitioners | Form on Website |  | Organization Contact |
| Three Rivers Chapter TAFP | Email |  | Organization Contact |
| TNA District 9 | Email | Email blast to members | Organization Contact |
| Travis County Medical Society | Email |  | Organization Contact |
| Treasurer of Permian Basin Area PA Society | Email |  | Organization Contact |
| Treasurer of Texas Gulf Coast PA Society | Email |  | Organization Contact |
| Trustee of Texas Gulf Coast PA Society | Email |  | Organization Contact |
| TTUHSC ALUMNI ENGAGEMENT MANAGER | Email |  | School Contact |
| TTUHSC El Paso | Email |  | School Contact |
| TTUHSC El Paso | Email |  | School Contact |
| Tx Institute for Graduate Med Ed and Research, Laredo | Email |  | School Contact |
| Tx Institute for Graduate Med Ed and Research, San Antonio | Email |  | School Contact |
| UT at Austin Fam and Comm Medicine Residency | Email |  | School Contact |
| UT at Tyler, Fam Med Res, Health Science Center, Athens | Email |  | School Contact |
| UT Dell Medical School | Email |  | School Contact |
| UT Health Center at Tyler | Email |  | School Contact |
| UT McGovern Medical School | Email |  | School Contact |
| UT McGovern Medical School | Email |  | School Contact |
| UT Southwestern | Email |  | School Contact |
| UT Southwestern Website | Email |  | School Contact |
| UT Tyler Health Science Center | Email |  | School Contact |
| UTMB | Email |  | School Contact |
| UTRGV | Email |  | School Contact |
| UTRGV Fam Med Residency at Doctors Hospital at Renaissance | Email |  | School Contact |
| UTRGV Fam Med Residency at Knapp Medical Center | Email |  | School Contact |
| UTRGV Fam Med Residency at McAllen Med Center | Email |  | School Contact |
| UTRGV Internal Med Residency at Doctors Hosp Renaissance | Email |  | School Contact |
| UTRGV Internal Med Residency at Knapp Med Ctr | Email |  | School Contact |
| UTRGV Internal Med Residency at Valley Baptist Med Ctr | Email |  | School Contact |
| UTRGV Obstetrics and GYN at Drs. Hosp at Renaissance | Email |  | School Contact |
| UTRGV Psychiatry Residency | Email |  | School Contact |
| UTRGV Transitional Year Residency at DHR | Email |  | School Contact |
| UTRGV-DHR | Email |  | School Contact |
| UTSA Family and Comm Medicine Residency | Email |  | School Contact |
| Valley Baptist Med Ctr (RGV) | Email |  | School Contact |
| Valley Chapter TAFP | Email |  | Organization Contact |
| Vice President of Permian Basin Area PA Society | Email |  | Organization Contact |
| Waco Chapter of TAAP | Email |  | Organization Contact |
| Wichita County Medical Alliance | Email |  | Organization Contact |
| Federation of Texas Psychiatry | Email | Advertisement in Newsletter | Organization Contact |
| Texas Association of Addiction Professionals | Email | Email blast to members | Organization Contact |
| Texas Tech Center of Excellence for Integrative Health | Email |  | Organization Contact |
| Bell County, Concho Valley, and Walter-Madison-Trinity County Medical Societies | Email |  | Organization Contact |
| Bexar County Medical Society | Email |  | Organization Contact |
| Brazos-Robertson County Medical Society | Email |  | Organization Contact |
| Collin-Fannin County Medical Society | Form on Website |  | Organization Contact |
| Dallas County Medical Society | Email |  | Organization Contact |
| Denton County Medical Society | Email |  | Organization Contact |
| Ector County Medical Society | Email |  | Organization Contact |
| El Paso Medical Society | Email |  | Organization Contact |
| Galveston County Medical Society | Email |  | Organization Contact |
| Guadalupe County Medical Society | Email |  | Organization Contact |
| Lubbock CMS | Email |  | Organization Contact |
| McLennan County Medical Society | Form on Website |  | Organization Contact |
| Montgomery County Medical Society | Email |  | Organization Contact |
| San Antonio TAFP | Email |  | Organization Contact |
| National Association of Social Work, Texas Chapter | Email | Email blast to members | Organization Contact |
| Potter-Randall CMS | Email |  | Organization Contact |
| Smith County Medical Society | Email |  | Organization Contact |
| Tarrant County CME | Email |  | Organization Contact |
| Tri County Medical Society | Email |  | Organization Contact |
| Wichita CMS | Email |  | Organization Contact |
| Williamson CMS | Email |  | Organization Contact |
| UTHealth Houston Cizik School of Nursing Alumni | Email |  | School Contact |
| Texas Women’s University Alumni | Email |  | School Contact |
| Texas A&M Nursing Alumni | Email |  | School Contact |
| UT Tyler Health Science Center alumni | Email |  | School Contact |
| Prairie View A&M University Alumni (nursing) --> specify nursing | Email |  | School Contact |
| University of Houston Alumni (Nursing) | Email |  | School Contact |
| West Texas A&M University College of Nursing (alumni) | Email |  | School Contact |
| Texas A&M Rural Medicine | Email |  | School Contact |
| Texas Academy of Physician Assistants (TAPA) | Email | Quarter Page advertisement in conference program | Organization Contact |
| Texas Substance Use Symposium | Email | Presentation | Organization Contact |
| 2022 Conference on Addiction, Recovery, and Families | Email | Flyer in welcome bag | Organization Contact |
| Texas Association for Marriage and Family Therapy (TMFT) | Email | Email sent to members | Organization Contact |
| Texas Counseling Association | Email | Ad on eUpdate | Organization Contact |
| Top of Texas PA Society | Email | Email to members | Organization Contact |
| BCM E-Rounds | Email | Notice in E-Rounds Newsletter | School Contact |
