# Supplementary material for: Factors associated with intention to implement SBI and SUD treatment: a survey of primary care clinicians in Texas enrolled in an online course
Source: BMC Prim Care. 2024 May 28;25:192. doi: 10.1186/s12875-024-02427-z (PMC11134618; doi:10.1186/s12875-024-02427-z)
Supplement: Supplementary file 2 — Supplementary Material 2 [file 12875_2024_2427_MOESM2_ESM.docx]

**Supplemental Table 2.** Comparisons of knowledge by clinician type among Texas primary health care clinicians (N=645)

| **Knowledge questions comparing correct to incorrect** | Family Medicine aOR (95% CI) ^a,b^ | Internal Medicine aOR (95% CI) ^a,b^ |
| --- | --- | --- |
| Advice you would give to pregnant patient about how much alcohol is safe during pregnancy ^a^ | **1.74 (1.12-2.73)** | 1.30 (0.70-2.42) |
| Which is considered a standard drink ^a^ | **1.75 (1.12-2.74)** | 1.32 (0.71-2.42) |
| Risky drinking in 21-yr old non-pregnant women (per week) ^a^ | **1.79 (1.14-2.81)** | 1.39 (0.75-2.58) |
| Evidence-based screening tool(s) used for SUD in primary care ^a^ | **1.66 (1.05-2.61)** | 1.35 (0.72-2.53) |
| First-line medication to treat tobacco cessation ^a^ | **1.66 (1.05-2.61)** | 1.25 (0.67-2.32) |
| Validated evidence-based screen for adolescent substance use ^a^ | **1.73 (1.10-2.72)** | 1.29 (0.69-2.41) |
| **Knowledge/ Attitude questions comparing attitudes consistent with evidence-based recommendations to those not consistent** |  |  |
| There is good evidence that primary care physicians can use brief interventions to decrease alcohol use in patients who drink at excessive levels. | **1.73 (1.11-2.71)** | 1.32 (0.71-2.45) |
| If a patient fails to respond to acamprosate to achieve abstinence from alcohol, naltrexone will show no benefit and should not be used. | **1.75 (1.11-2.74)** | 1.36 (0.73-2.53) |
| Opioid cravings can be treated in an office setting with buprenorphine. | **1.72 (1.1-2.70)** | 1.30 (0.70-2.42) |
| A local pharmacist contacts you because one of your patients has also been receiving prescriptions for hydrocodone from two other doctors. The most appropriate management is to tell the pharmacist to cancel the prescription and to discharge the patient from your practice. | **1.77 (1.13-2.77)** | 1.30 (0.70-2.41) |
| A 34-year-old patient is not ready to stop drinking. The most appropriate next step, using motivational interviewing, is to forcefully confront him with the likely health consequences of continued alcohol use. | **1.71 (1.09-2.68)** | 1.30 (0.70-2.42) |
| A person is more likely to be successful if they focus on quitting both alcohol and nicotine at once rather than one at a time. | **1.74 (1.11-2.72)** | 1.31 (0.70-2.42) |

^a^Family medicine and Internal medicine practitioners compared to “other” types of practitioners, which included: addiction medicine, obstetrics/gynecology, pediatrics, psychiatry, preventive/occupational, and sports medicine.

^b^Analyses controlled for gender. aOR = adjusted odds ratio and 95% CI = 95% confidence interval.
